# Left Atrial Myxoma Embolization Presenting as Acute Limb Ischemia: An Unusual Presentation

**DOI:** 10.7759/cureus.2764

**Published:** 2018-06-08

**Authors:** Mohamed A Mohamed, Anan Tawil, Mohammed Al Salihi, Mark Mattos

**Affiliations:** 1 Michigan State University College of Human Medicine; 2 Vascular Surgery, Michigan Vascular Center; 3 Internal Medicine, Hurley Medical Center, Michigan State University

**Keywords:** acute limb ischemia, atrial myxoma, vascular disease, cardiac neoplastic tumors

## Abstract

Cardiac myxomas are rare with reported incidences of less than 0.03%. Cardiac myxomas are most commonly observed in the left atrium. Their clinical manifestations vary and most are non-specific to the diagnosis. The most common extra-cardiac manifestations are thrombo-embolic infarcts from tumor embolization. A previously healthy 55-year-old man presented with findings suggestive of acute arterial limb ischemia. Following surgical treatment of his acute presentation, a left atrial mass was found on echocardiography suggesting that the embolization was secondary to a cardiac myxoma. The patient was discharged without complication. Embolic myxoma should be included in the differential in younger, previously healthy patients presenting with acute arterial limb ischemia without obvious cause. Our patient’s dramatic presentation with a single acute event, however, prompted immediate treatment and resulted in a functional recovery with minimal complications.

## Introduction

Cardiac myxomas (CMs) are the most common primary cardiac neoplasms, accounting for 50% of all cardiac tumors. These benign tumors are rare, with reported incidences of less than 0.03% [1]. The vast majority (80%) of CMs are left atrial myxomas (LAMs), although CMs can occur in any cardiac chamber [2-3]. Up to 50% of LAMs may result in systemic embolization. Moreover, the secondary manifestations of LAMs may mimic other more common conditions such as a vasculitis, which may make the diagnosis difficult [2]. LAM embolization presenting as acute limb ischemia (ALI) is rarely reported. Herein, we present an unusual case of LAM embolization resulting in ALI in a previously healthy young man. 

## Case presentation

A 55-year-old previously healthy man presented with a two-hour history of abrupt, severe pain and paresthesia of the right lower extremity (RLE) after playing basketball. This is the first time he had experienced this, but he had experienced intermittent episodes of dizziness and palpitations over the past two months. He denied any history of trauma, smoking, alcohol abuse, and illicit drug use. 

**Figure 1:**
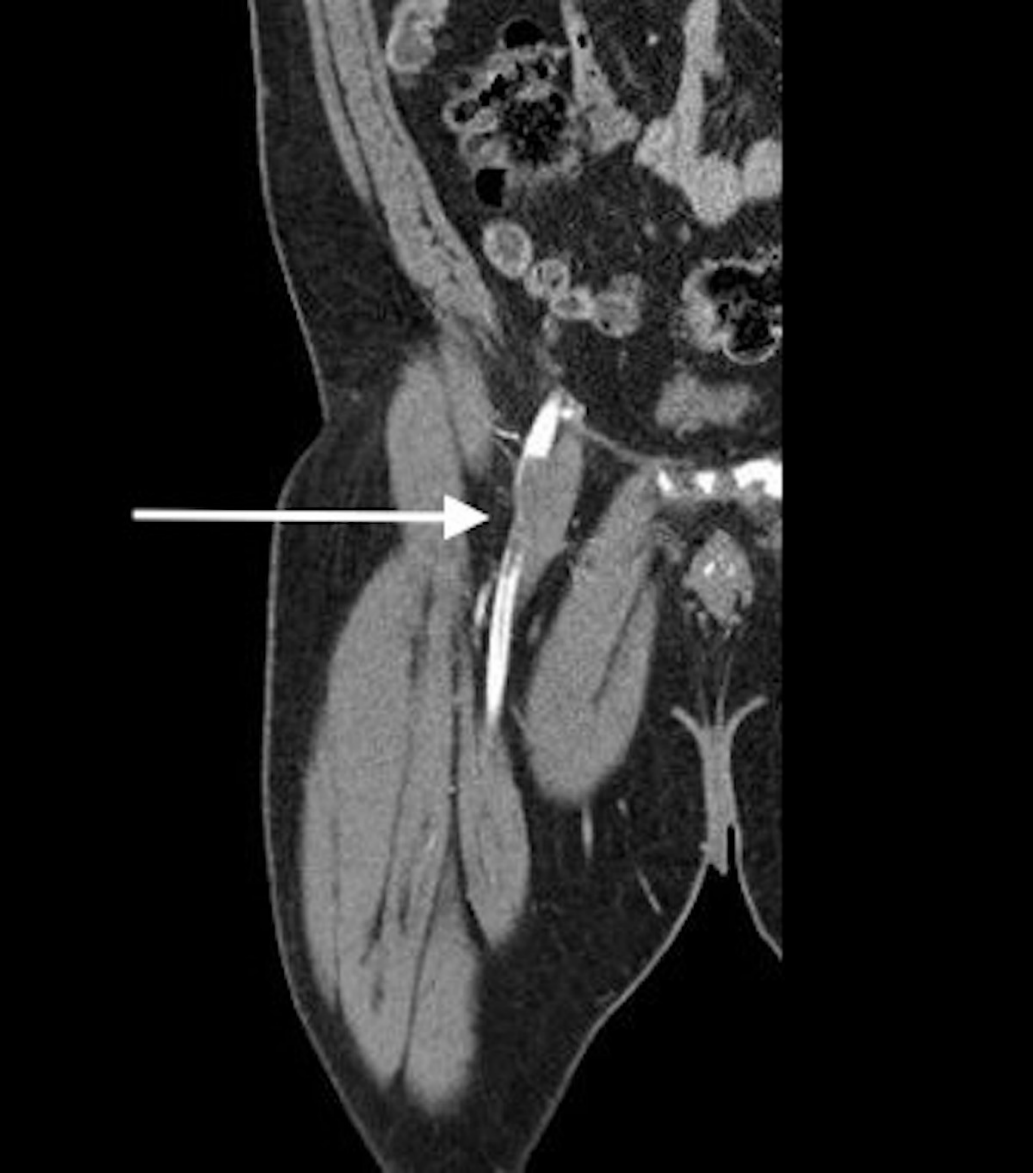
Computed tomography with contrast (frontal plane) of left lower extremity demonstrating occlusion of the common femoral artery.

**Figure 2:**
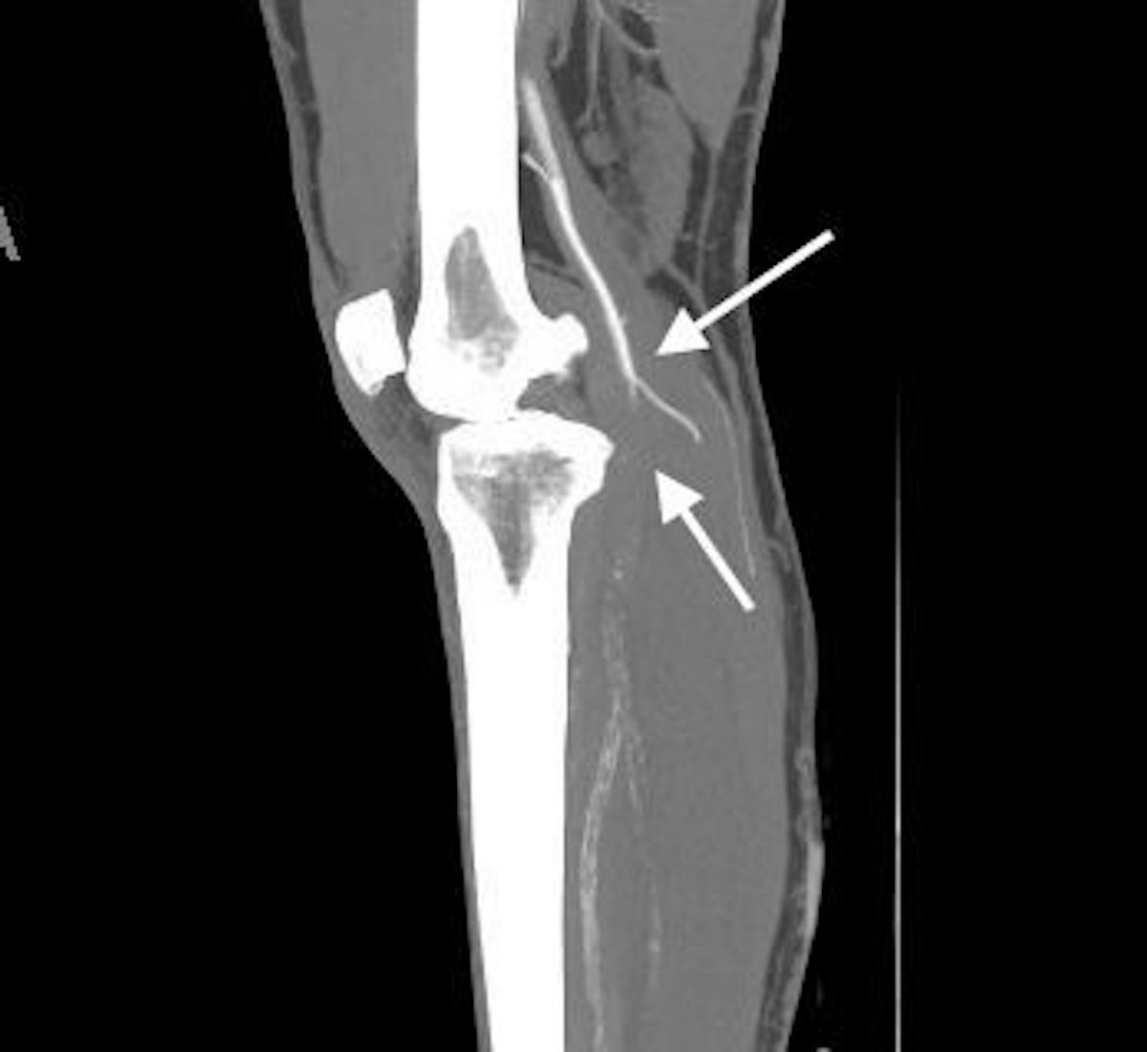
Computed tomography with contrast (sagittal plane) of left lower extremity demonstrating occlusion of the proximal popliteal artery.

Vitals were as follows: blood pressure (BP) from the right arm - 151/83 mmHg; heart rate - 102 beats/min; respiratory rate - 16 breaths/min; temperature - 36.3° C, and oxygen saturation - 100% on room air. On examination, his RLE was cold, pale, and insensate. There were no palpable femoral, popliteal, or pedal pulses in the RLE. Distal pulses were also negative by Doppler ultrasound. He had conserved 5/5 motor strength in both feet. Examination of the left lower extremity (LLE) was normal. Cardiopulmonary exam was unremarkable, with no murmurs or audible extra heart sounds. All other physical exam and laboratory findings were unremarkable. Computed tomography angiography (CTA) of the RLE revealed an occlusion of the right common femoral artery (CFA). There was also a second occlusion at the proximal popliteal artery at the level of the knee joint with no opacification of the leg vasculature distal to the popliteal artery (Figures 1-2). Electrocardiogram did not reveal any atrial fibrillation.

The patient was diagnosed with ALI secondary to an arterial embolus of unknown origin initially, started on heparin, and was taken emergently to the operating room for right groin exploration and a femoral thromboembolectomy by groin cut-down with a four-compartment fasciotomy. An intraoperative angiogram of the RLE showed good runoff and no additional occlusion distally. A formal arteriogram of the aorta was not performed. The artery was closed, and a four-compartment fasciotomy was performed on the right lower leg. The leg showed evidence of reperfusion ischemia. In the recovery room, the patient had palpable pedal pulses in both legs and heparin anticoagulation was continued.

**Figure 3:**
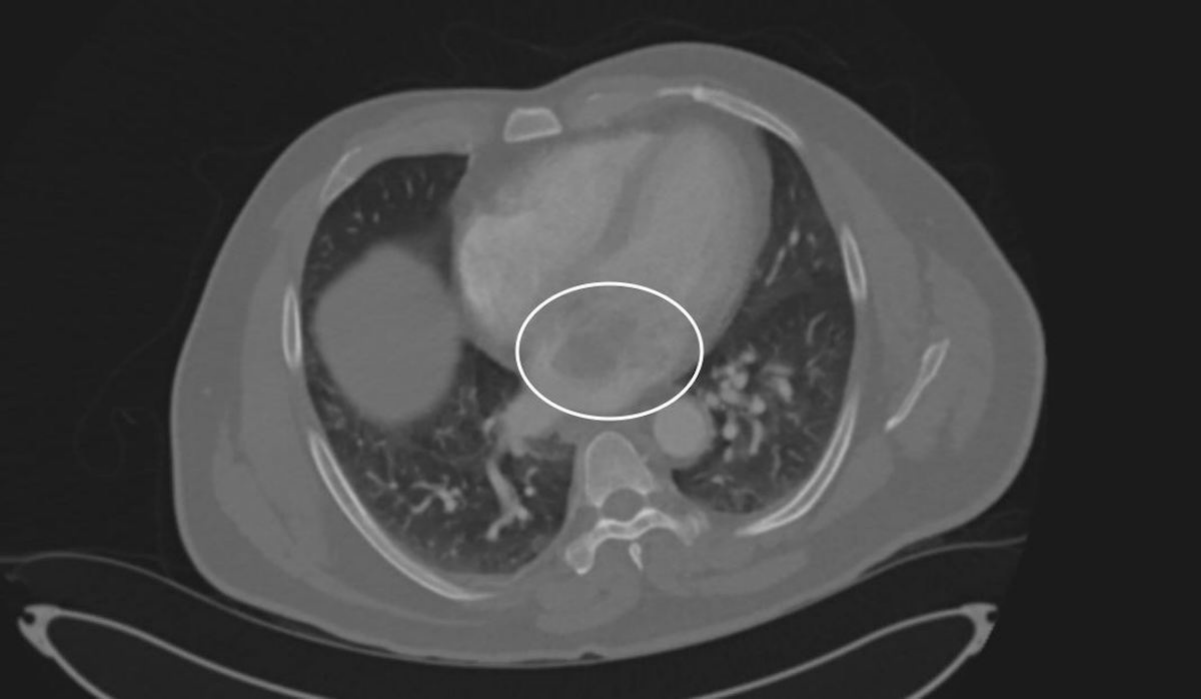
Computed tomography with contrast of chest (Transverse view) demonstrating a soft tissue mass in the left atrium.

**Figure 4:**
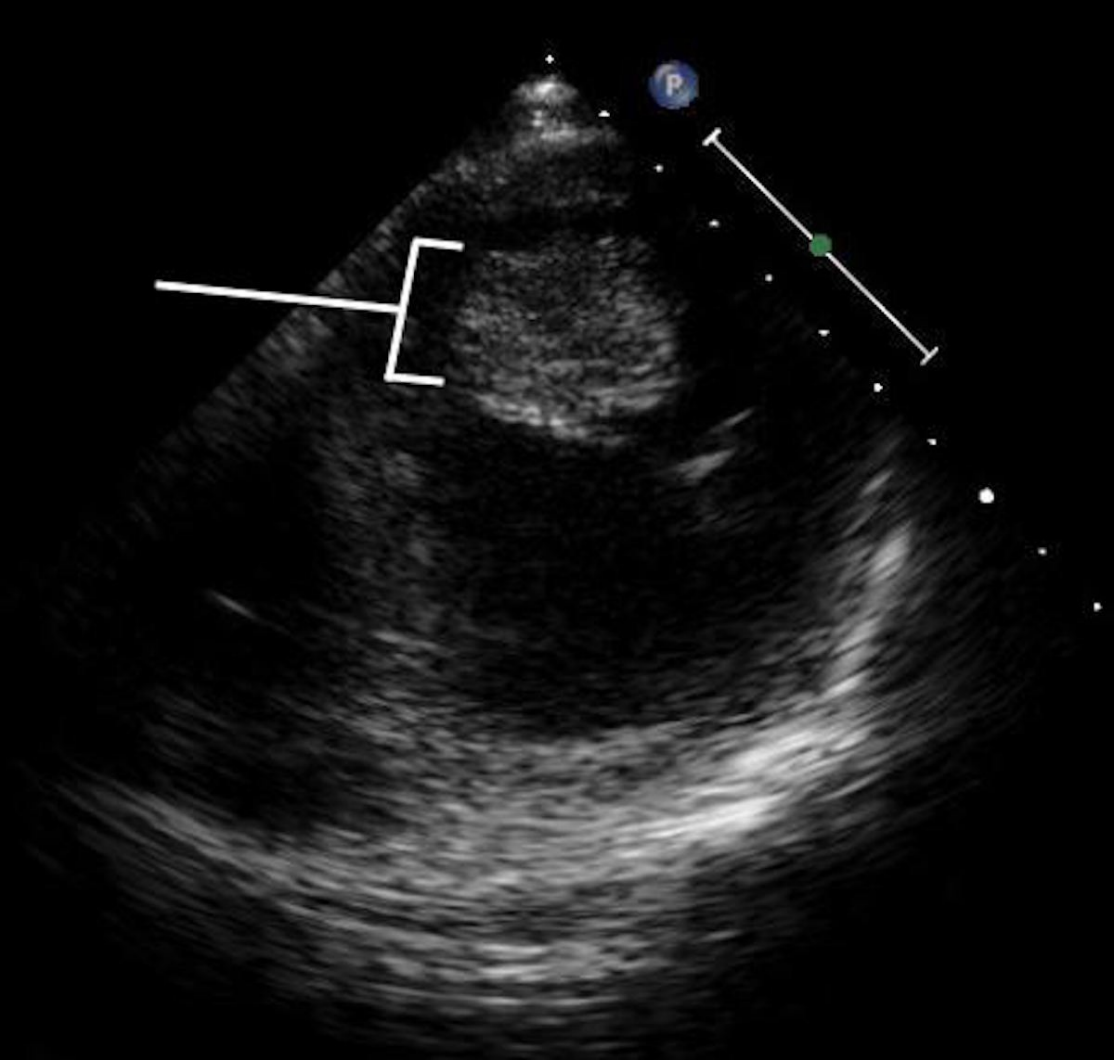
Transesophageal echocardiogram showing the myxoma attaching to the interatrial septum.

On postoperative day one (POD 1), the patient’s troponin and creatinine kinase levels were elevated. A CTA of the chest revealed an irregular mass in the left atrium (Figures 1-3), which was also observed on a subsequent echocardiogram which also showed that the mass was attached to the interatrial septum and was bulging through the mitral valve (Figure 4). The patient’s fasciotomies were closed on POD 6, and he was discharged from the hospital on warfarin without further complication. Histological evaluation of the embolus revealed spindle-shaped cells set within a collagenous myxoid stroma, which definitively confirmed the diagnosis. The patient was sent to a tertiary center for further managment by cardiothoracic surgery.

## Discussion

Myxomas arise from mesenchymal stem cell dysregulation resulting in over-proliferation. These stem cells have the capacity to differentiate into smooth muscle cells, fibroblasts, and endothelial cells, whose roles are vital in the cardiovascular system [3-4]. Histologically, CMs are often described as lipidic cells embedded in a myxoid vascular stroma. They arise from the subendocardium surrounded by a thin capsule of remnant endocardial tissue. Furthermore, CMs are often pedunculated, most commonly attaching to the fossa ovalis in the LA via a thin stalk of fibroelastic tissue [5].

The majority of CMs are sporadic, but up to 7% of CMs are familial with an autosomal dominant inheritance pattern with multiple families being linked to chromosome 2p16 [5-6]. Although LAMs are benign, severe and even fatal outcomes have been reported and are not infrequent. These rare tumors are frequently observed in the fourth and fifth decades of life and are slightly more prevalent in women, though they occur in all age groups and genders [7].

The clinical manifestations of LAMs are heterogeneous and up to 20% of patients are completely asymptomatic [2]. LAM presentations depend on multiple factors including size, shape, and its anatomical location. The majority of LAMs present with symptoms secondary to an obstructive pattern. Auscultation often mimics mitral stenosis with a loud S1 due to delayed mitral valve closure from LAM prolapse. Additionally, auscultation often reveals a “tumor plop” due to the “wrecking ball” effect of the pedunculated tumor as it moves back and forth between the atrium and the ventricle [8].

The pathophysiology by which the penduculated LAMs arise make them very friable and therefore susceptible to detachment. Consequently, almost half of all LAMs result in peripheral embolization with the cerebral circulation being the most common site of embolization, followed by the pulmonary and coronary vasculature [2]. In theory, tumor size may be an important prognostic factor in these patients, with a size greater than 4.5 cm observed to be an independent risk factor for embolization. Although smaller CMs may embolize more frequently than larger CMs, this phenomenon is still rarely observed. Moreover, larger CMs are more favorable for thrombus formation and thromboembolization, although this phenomenon can occur with all sizes [9].  

Laboratory findings suggesting inflammatory patterns are well reported in cases of CMs and have been associated with anemia and increased expression of interleukin-6, and elevated troponins and globulin can also be observed [8]. A 28-year study reported that 74% of CMs also had elevated inflammatory markers, erythrocyte sedimentation rate (ESR), and C-reactive protein (CRP), which may explain the female predominance of the condition [6]. The mechanism behind this is unclear. Additionally, because patients may only present with constitutional symptoms and elevated inflammatory markers, several cases in our literature review were misdiagnosed as an autoimmune vasculitis.

Echocardiography is the mainstay of diagnosis with histological evaluation for definitive confirmation. To our knowledge, there is no evidence of conservative medical management to decrease tumor size or for complete ablation [9]. Treatment of LAMs is through surgical resection and/or total excision with debridement of some of the healthy tissue, both of which are associated with excellent long-term survival.

Recurrence has been reported in up to 5% of sporadic CMs and up to 20% in familial cases and is more likely within the first 10-years postoperatively [8-9]. Thus, follow-up monitoring is required during the first decade following resection. Recurrence has been observed at the original implantation site, at adjacent locations such as the lungs, and at distant sites as a result of embolization. Malignant transformation has also been described [1].

## Conclusions

Acute arterial limb ischemia secondary to a cardiac myxoma is rarely reported, but our literature review suggests that it is not infrequent. A high index of suspicion should be prompted when younger patients present with acute arterial limb ischemia in the setting of constitutional symptoms or obstructive manifestations in the absence of known cardiovascular risk factors. Echocardiography is standard for diagnosis, although a definitive diagnosis is only arrived at through histology. Timely diagnosis is vital, and outcomes are favorable with prompt surgical management. Our patient’s dramatic presentation with a single acute event prompted immediate treatment and resulted in functional recovery with minimal complications.
